# Pembrolizumab-associated minimal change disease in a patient with malignant pleural mesothelioma

**DOI:** 10.1186/s12885-016-2718-y

**Published:** 2016-08-19

**Authors:** Angelika Bickel, Irene Koneth, Annette Enzler-Tschudy, Jörg Neuweiler, Lukas Flatz, Martin Früh

**Affiliations:** 1 Department of Oncology and Haematology, Cantonal Hospital St. Gallen, Rorschacherstrasse 95, 9007 St. Gallen, Switzerland; 2 Division of Nephrology and Transplantation Medicine, Cantonal Hospital St.Gallen, St. Gallen, Switzerland; 3 Institute of Pathology, Cantonal Hospital St. Gallen, St. Gallen, Switzerland; 4 Department of Dermatology, Cantonal Hospital St. Gallen, St. Gallen, Switzerland

**Keywords:** Malignant Pleural Mesothelioma, PD-1 antibody, Pembrolizumab, Immunotherapy, Minimal change disease

## Abstract

**Background:**

Pembrolizumab is an anti- Programmed Death 1 (PD-1) antibody approved in melanoma, non-small cell lung cancer and investigated in malignant pleural mesothelioma.

The most frequent immunotherapy related autoimmune reactions include dermatitis, pneumonitis, colitis, hypophysitis, uveitis, hypothyreodism, hepatitis and interstitial nephritis.

**Case presentation:**

We describe a 62-year old patient diagnosed with malignant pleural mesothelioma who experienced ten days after the second dose of third line therapy with pembrolizumab sudden onset of generalized edema including legs and eyelids and weight gain of 15 kg resulting from nephrotic syndrome and acute renal failure. Pembrolizumab was discontinued and prednisone, diuretics and angiotensin II receptor blocker were initiated with full recovery of symptoms and renal function. Pembrolizumab-associated minimal change disease (MCD) was confirmed by electron microscopy in the renal biopsy.

**Conclusion:**

We are the first to describe pembrolizumab-related minimal change disease (MCD).

Physicians should be aware of this side effect in patients presenting with edema and weight gain and initiate prompt renal function testing, serum albumin and urinalysis followed by steroid treatment if pembrolizumab-related MCD is suspected.

## Case presentation

**Fig. 1:**
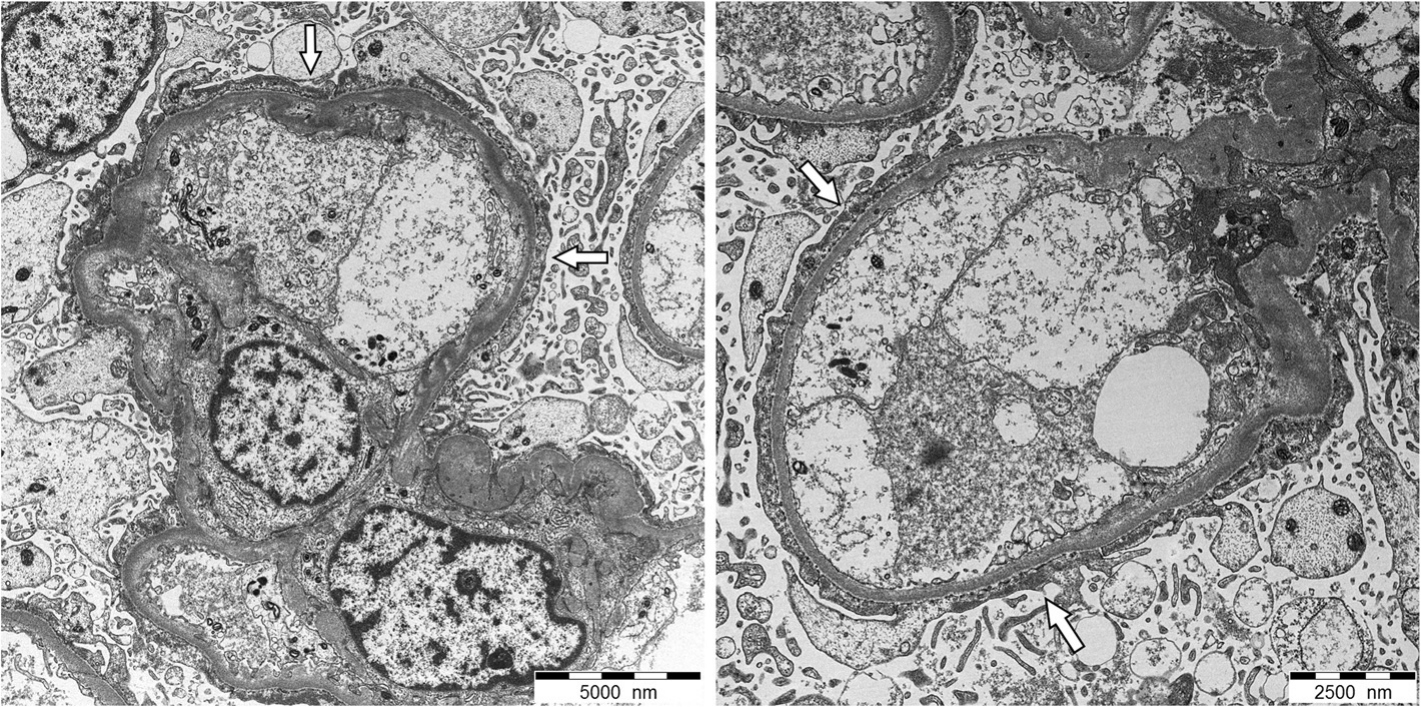
Electron microscopy. Glomerular capillary loops with findings of minimal change disease: Podocytes with extensive, diffuse foot process effacement (with arrows) and microvillous transformation. No electron-dense deposits. Normal thickness of glomerular basement membrane. Uranyl acetate and lead citrate

A 62-year old patient was diagnosed with malignant biphasic pleural mesothelioma in June 2014. He experienced progressive disease after six cycles of carboplatin/pemetrexed and six cycles of second line vinorelbine. Ten days after the second dose of 200 mg of a third line therapy with pembrolizumab sudden onset of generalized edema including the legs and eyelids with weight gain of 15 kg occurred. Nephrotic syndrome with proteinuria of 19 g per day, hypoalbuminemia (15 g/l) and hypercholesterolemia of 7.8 mmol/l was diagnosed followed by acute renal failure with a rapid deterioration of the estimated glomerular filtration rate from > 90 to 28 ml/min/1.73 m^2^. Pembrolizumab was discontinued due to suspected immune-related renal toxicity and prednisone (1 mg/kg/ day), diuretics and an angiotensin II receptor blocker was initiated. A renal biopsy showed diffuse fusions of the epithelial foot processes on electron microscopy compatible with minimal change disease (MCD) (Fig. 1). Direct immunofluorescence microscopy was negative with no complement or immunoglobulin deposits. Within 5 weeks, creatinine values normalized and the proteinuria resolved. The patient reported on symptomatic relief of disease-associated chest pain and currently remains in stable condition without systemic therapy 10 weeks later.

## Background and Conclusion

Pembrolizumab is an anti- Programmed Death 1 (PD-1) antibody approved for advanced melanoma that progressed following ipilimumab and, if BRAF[V600] mutant, a BRAF inhibitor and for PD-L (ligand) 1 positive advanced non-small cell lung cancer after progression to standard platinum based firstline treatment. It is investigated in multiple cancers including malignant pleural mesothelioma. Preliminary results of a single arm trial with 25 pretreated PD-L1- positive mesothelioma patients demonstrated an encouraging response rate of 28 %, a disease control rate of 76 % including durable response rates and a progression free survival rate at 6 months of 49 % [1]. Pembrolizumab disrupts the engagement of PD-1 with its ligands and impedes inhibitory signals leading to recognition of tumor cells by cytotoxic T cells. Immune related-adverse events such as dermatitis, hypophysitis, colitis and hepatitis have been reported for this class of agents. Whereas interstitial nephritis is a rare, but a well-recognised serious renal side effect, MCD has not been described in this context. Early trials with pembrolizumab have reported grade 3 and 4 edema in two patients [2] without further specification of the underlying cause. In our patient, clinical signs of nephrotic syndrome with hypovolemic acute renal failure and histologic findings on electron microscopy confirmed the diagnosis. MCD mainly occurs in conditions with activated adaptive immune system such as in young children, hematologic malignancies and autoimmune diseases. The pathology of MCD is poorly understood. Alterations of regulatory T-cells or their differentiation, epigenetic mechanisms and up-regulated secretion of proteins by podocytes may play an etiologic role. Typically, no specific changes are notable on light microscopy (ie the term “minimal change”), electron microscopy almost always shows loss of podocytes or at least a change of podocyte architecture. MCD has also recently been described in a patient receiving ipilimumab and in mesothelioma patients in the absence of immunotherapy [3, 4]. Whether a structural and functional similarity of podocytes and mesothelial cells increases the risk of MCD with immunotherapy in this particular patient population would have to be further investigated.

To the best of our knowledge, this is the first case of a pembrolizumab- associated MCD resulting in nephrotic syndrome and acute renal failure.

## Abbreviations

MCD: Minimal change disease
PD-1: Programmed death 1
